# An Integrated Method Based on PSO and EDA for the Max-Cut Problem

**DOI:** 10.1155/2016/3420671

**Published:** 2016-02-18

**Authors:** Geng Lin, Jian Guan

**Affiliations:** ^1^Department of Mathematics, Minjiang University, Fuzhou 350108, China; ^2^Modern Educational Technology Center, Minjiang University, Fuzhou 350108, China

## Abstract

The max-cut problem is NP-hard combinatorial optimization problem with many real world applications. In this paper, we propose an integrated method based on particle swarm optimization and estimation of distribution algorithm (PSO-EDA) for solving the max-cut problem. The integrated algorithm overcomes the shortcomings of particle swarm optimization and estimation of distribution algorithm. To enhance the performance of the PSO-EDA, a fast local search procedure is applied. In addition, a path relinking procedure is developed to intensify the search. To evaluate the performance of PSO-EDA, extensive experiments were carried out on two sets of benchmark instances with 800 to 20000 vertices from the literature. Computational results and comparisons show that PSO-EDA significantly outperforms the existing PSO-based and EDA-based algorithms for the max-cut problem. Compared with other best performing algorithms, PSO-EDA is able to find very competitive results in terms of solution quality.

## 1. Introduction

The max-cut problem is one of the most classical combinatorial optimization problems. It is formally defined as follows. Given an undirected graph *G*(*V*, *E*), with vertices set *V* = {1,…, *n*} and edges set *E*, each edge (*i*, *j*) ∈ *E* being associated with a weight *w*_*ij*_, the max-cut problem is to find a partition (*V*_1_, *V*_2_) of *V*, so as to maximize the sum of the weights of the edges between vertices in the different subsets.

Let *x* = (*x*_1_,…, *x*_*n*_)^*T*^ ∈ {1, −1}^*n*^ denote a solution of the max-cut problem. *x*_*i*_ = 1  (*x*_*i*_ = −1) indicates that vertex *i* is partitioned into *V*_1_  (*V*_2_). Let *W* = (*w*_*ij*_)_*n*×*n*_ be the symmetric weighted adjacency matrix of *G*. The max-cut problem can be formulated as the following discrete quadratic optimization problem [1]:

 (MC)
  (1) max⁡ fx=xTL^xs.t. xi∈1,−1,i∈1,…,n,

where $\hat{L}=Diag(We)-W$ is the Laplace matrix of *G*.

The max-cut problem has long served as a challenging test for researchers testing new methods for combinatorial algorithms [2] and has a wide range of practical applications such as numerics, scientific computing, circuit layout design, and statistical physics. It is one of the Karp's original NP-complete problems [3].

Due to the significance of the max-cut problem in academic research and real applications, it has gained much attention over the last decade. Because of the NP-hardness of the max-cut problem, heuristics have a crucial role for the solution of large scale instances in acceptable computing time. Various heuristic methods have been proposed including rank-two relaxation heuristic [4], GRASP [5, 6], scatter search [7], filled function method [1], dynamic convexized method [8], tabu Hopfield network and estimation of distribution [9], tabu search [2], particle swarm optimization [10], path relinking [11], breakout local search [12], and tabu search based hybrid evolutionary algorithm [13]. Among the above heuristic algorithms, breakout local search, path relinking, and tabu search based hybrid evolutionary algorithm are the best heuristics for solving challenging max-cut problems.

Particle swarm optimization (PSO) [14] is one of the most popular population-based algorithms. In this technique, all particles search for food in the search space based on their positions and velocities. Every particle can adjust its flying direction by learning from its own experience and the performance of its peers [15]. Different variants of PSO have been shown to offer good performance in a variety of continuous and discrete optimization problems [16, 17]. Although information between particles is shared with each other to some extent, it is performed in a strictly limited fashion, and the global information about the search space is not utilized.

Estimation of distribution algorithm (EDA) [18] is a new paradigm in the field of evolutionary computation and has been applied to solve many optimization problems [19–21]. It uses a probability model, which gathers the global information of the search space, to generate promising offsprings. The probability model is updated at each generation using the global statistical information. However, the local information of the solutions found so far is not utilized. The algorithm may get stuck at local optima due to lack of diversity.

Blum et al. [22] observed that complementary characteristics of different optimization heuristics benefit from hybridization; for example, see [23, 24]. In this work, we focus on developing an integrated algorithm (PSO-EDA) based on PSO and EDA to benefit from the advantages of PSO and EDA. The integrated algorithm PSO-EDA consists of hybridization of PSO, EDA, local search procedure, path relinking, and mutation procedure. PSO is utilized to find local information of the search space quickly, while EDA is used to guide the search by the global information. Local search procedure and path relinking are applied to further improve the solution quality. To maintain the diversity, mutation procedure is adopted. The integrated algorithm overcomes the shortcomings of PSO and EDA and keeps a proper balance between diversification and intensification during the search. We use two sets of 91 benchmark instances from the literature to test the performances of the PSO-EDA. The comparisons of PSO-EDA with the existing PSO-based and EDA-based algorithms for the max-cut problem show that PSO-EDA significantly outperforms these algorithms in terms of solution quality and solution time. Compared with other metaheuristic algorithms, including GRASP, breakout local search, path relinking, and tabu search based hybrid evolutionary algorithm, PSO-EDA can find very competitive results in terms of solution quality. Moreover, PSO-EDA finds the best known solutions on 62 instances out of total 91 instances. In addition, its deviations range from 0.01% to 0.47%. It shows that the proposed algorithm is able to find high quality solutions of the max-cut problem.

The remainder of this paper is organized as follows.  Section 2 describes a detailed explanation of the PSO-EDA. The computational results and comparisons are given in Section 3. The conclusion remarks are made in Section 4.

## 2. The Proposed Algorithm

### 2.1. The Framework of PSO-EDA

The general structure of the PSO-EDA is given in Algorithm 1. Essentially, PSO-EDA alternates between PSO procedure and EDA procedure. PSO procedure and EDA procedure play different roles in PSO-EDA. PSO procedure is used to gather the local information. The obtained local information is then used to update the probability vector while EDA procedure is used to guide the search by the global information. It generates new promising solutions. These two complementary procedures are iteratively performed to obtain high quality solutions.

In the PSO-EDA, (*n* − 1)-dimensional probability vector *p* = (*p*_2_,…, *p*_*n*_)∈[0,1]^*n*−1^ is used to represent the probability model of the solution space, where *p*_*j*_  (*j* = 2,…, *n*) is the probability of *x*_*j*_ = *x*_1_. Let *g* be the current generation. At the beginning of PSO-EDA, a population Pop(*g*) consisting of *s* particles is generated randomly, and each particle is further improved by a local search procedure. Let ${\hat{x}}^{i}$ be the personal best position of particle *i*, and let *x* ^*∗*^ be the best solution found so far. We initialize ${\hat{x}}^{i}=x^{i}$ and *x* ^*∗*^ = arg⁡max⁡{*f*(*x* ^*i*^), *i* = 1,…, *s*.}. The probability vector is initialized as *p* = (0.5,…, 0.5). Then, PSO procedure and EDA procedure are executed alternately. If *g* can be divided by 2, EDA procedure is performed; otherwise, PSO procedure is executed. After that, a new population Pop′(*g*) is generated and is used to form the next population Pop(*g* + 1). If the current best solution *x* ^*∗*^ is not improved after *G*_no_ continuous generations, the current personal best solutions can not guide the search efficiently. Each ${\hat{x}}^{i}$ is perturbed by a mutation procedure and improved by the local search procedure. The obtained solution is used to replace the personal best solution of particle *i*. The above process is repeated until Maxcount of generations is reached.

The PSO-EDA consists of five main components: PSO procedure, EDA procedure, local search procedure, path relinking procedure, and mutation procedure. These procedures are described in detail in the following subsections.

### 2.2. PSO Procedure

The standard PSO is introduced for solving continuous optimization problems. To deal with discrete optimization problems, Kennedy and Eberhart [26] developed a binary version of PSO. After that, many discrete versions of PSO [27–29] have been proposed. Recently, Qin et al. [28] proposed an algorithmic framework of discrete PSO (denoted by DPSO for short), and the application of DPSO to number partitioning problem has demonstrated the effectiveness of the proposed algorithm.

The basic idea of canonical PSO is that any particle moves close to the best of its neighbors and returns to the best position of itself so far. The DPSO follows the basic idea of canonical PSO. It uses one of the following equations to generate a new position for particle *i* in the swarm:

(2) xi=xi⊕r1•x^i~xi,

(3) xi=xi⊕r2•x^iN~xi,

(4) xi=xi⊕r3•xr~xi,

where ${\hat{x}}^{i}$ and ${\hat{x}}^{iN}$ are personal best position of particle *i* and the neighborhood best position, respectively; *r*_1_, *r*_2_, and *r*_3_ are three random numbers in [0,1]; *x* ^*r*^ is chosen at random from the current swarm; and ~, •, and ⊕ are three operators, and their definitions are as follows.

Difference operator (~): given any positions *x* and *y*, the difference of them, denoted by *x* ~ *y*, is a sequence of least number of consecutive flip operators. Difference of two positions is used to act as velocity in the DPSO; that is, *v* = *x* ~ *y*.

Product operator (•): supposing that *σ* is a real number and *v* is a velocity (i.e., the difference of two positions), the product of them, denoted by *σ*•*v*, is a subsequence of *v* such that the length of this subsequence is [*σ*|*v*|], where |*v*| is the length of *v*.

Sum operator (⊕): given a position *x* and a velocity *v*, *x* ⊕ *v* starts with *x* and flips all the variables in *v* to obtain a new position.

Equations (2) and (3) try to make particle moves close to the best position of itself so far and the best of its neighbors, respectively. Equation (4) introduces a stochastic factor to avoid premature convergence of DPSO. In each iteration, exactly one of the three equations is employed to update a particle.

Inspired by the idea in [28], our PSO procedure employs the basic structure of DPSO [28] and redefines the operators of DPSO based on the specific structure of the max-cut problem. Supposing that *x* = (*x*_1_,…, *x*_*n*_) and *y* = (*y*_1_,…, *y*_*n*_) are two solutions of max-cut problem, we define *x* ~ *y* = {*j*∣*x*_*j*_ ≠ *y*_*j*_, *j* ∈ {1,…, *n*}}. It is used to determine the differences between *x* and *y*. In our PSO procedure, the velocity *v* is denoted as a set of variables, that is, *x* ~ *y*. Different from the definition of operator • in DPSO, our operation *σ*•*v* generates a variable subset of *v* by removing each variable *j* ∈ *v* from *v* with a probability *σ*. This operator increases the exploration ability of our PSO procedure. The operation *x* ⊕ *v* generates a new solution. It starts from *x* and flips all the variables in *v*.

In (3), particle *i* tries to reduce the distance to the best of its neighbors ${\hat{x}}^{iN}$. It is time consuming to update ${\hat{x}}^{iN}$ for each particle *i* in each generation, especially for large scale problems. In addition, the landscape analysis of max-bisection problem [30] shows that, in most cases, the distances between high quality solutions are very small. Their research result [30] indicates that the degree of similarity between ${\hat{x}}^{iN}$ and the current best solution *x* ^*∗*^ is very large. To speed up the search, each particle tries to move close to *x* ^*∗*^. The search can concentrate fast around *x* ^*∗*^. In our PSO procedure, (3) is replaced by

(5) $$\begin{array}{c} x^{i}=x^{i}⊕\left(\;r_{2}•\left(\;x^{*}\sim x^{i}\;\right)\;\right). \end{array}$$

The pseudocode of our PSO procedure is given in Algorithm 2. For each solution *x* ^*i*^ in the population Pop(*g*), a new position is updated by (2), (5), and (4) with probabilities prob_*p*_, prob_*n*_, and prob_*r*_, respectively (lines (1)–(11)). We have prob_*p*_ + prob_*n*_ + prob_*r*_ = 1; that is, updating of a particle is influenced by exactly one of (2), (5), and (4). Then, the newly obtained position is further improved by a local search procedure (line (12)). We use a path relinking procedure, which will be described in Section 2.5, to intensify the search. And ${\hat{x}}^{i}$ and *x* ^*∗*^ are updated (line (13)).

### 2.3. EDA Procedure

EDAs produce offsprings through sampling according to a probability model. Probability models identify the remarkable features of promising candidate solutions from the population. A probability model has a great effect on the performance of EDA.

Notice that *x* = (*x*_1_,…, *x*_*n*_) and its symmetric solution $\bar{x}=(-x_{1},\ldots ,-x_{n})$ correspond to the same partition of *V*. Since the solution space of the max-cut problem is symmetric, the traditional probability models used in other binary optimization problems can not be directly applied.

We propose a probability model according to the symmetric solution space of the max-cut problem. Our EDA procedure uses (*n* − 1)-dimensional vector *p* = (*p*_2_,…, *p*_*n*_) to characterize the distribution of promising solutions in the search space, where *p*_*j*_  (*j* ≠ 1) is the probability of *x*_*j*_ = *x*_1_.

At the beginning of the PSO-EDA, vector *p* is initialized as *p* = (0.5,…, 0.5). The PSO-EDA performs the PSO procedure and EDA procedure alternately. After the PSO procedure, a new population Pop(*g*), which contains *s* new local optimal solutions, is obtained. The EDA procedure identifies the best *N* solutions in Pop(*g*), and the probability vector *p* ^*g*−1^ is updated according to the following equation:

(6) $$\begin{array}{c} p_{j}^{g}=\left(\;1-\alpha \;\right)p_{j}^{g-1}+\alpha \frac{1}{N}\overset{N}{\underset{k=1}{\sum }}\left|\;\frac{x_{j}^{k}+x_{1}^{k}}{2}\;\right|,\;j=2,\ldots ,n, \end{array}$$

where *p* ^*g*^ is the probability vector in the *g*th generation, *x* ^*k*^ = (*x*_1_^*k*^,…, *x*_*n*_^*k*^) is the *k*th individual of the best *N* solutions in Pop(*g*), and *α* ∈ (0,1) is the learning speed. Since *x*_*j*_^*k*^ ∈ {1, −1}, |(*x*_*j*_^*k*^ + *x*_1_^*k*^)/2| is binary. If *x*_*j*_^*k*^ = *x*_1_^*k*^, it holds |(*x*_*j*_^*k*^ + *x*_1_^*k*^)/2| = 1; otherwise, we have |(*x*_*j*_^*k*^ + *x*_1_^*k*^)/2| = 0.

Wu and Hao [30] concluded from their experimental tests that the degrees of similarity between high quality solutions are very large. The best *N* solutions, which are selected to update *p* ^*g*^, may be very similar. It leads the EDA procedure to produce very similar new solutions. The range of the value of probability *p*_*j*_^*g*^ is limited to an interval [*p*_min_, *p*_max_] with the aim of avoiding search stagnation. More formally, the probability vector *p* ^*g*^ is reset as follows:

(7) $$\begin{array}{c} p_{j}^{g}=\left\{\begin{array}{cc} p_{min} & \text{if }p_{j}^{g}<p_{min},\\ p_{j}^{g} & \text{if }p_{min}\leq p_{j}^{g}\leq p_{max}\\ p_{max} & \text{if }p_{j}^{g}>p_{max}. \end{array}\right.\;j=2,\ldots ,n, \end{array}$$

In each generation of the PSO-EDA, EDA procedure generates new solutions via sampling according to the probability vector *p* ^*g*^. A new solution *x* = (*x*_1_,…, *x*_*n*_) is generated as follows. First, EDA procedure randomly generates *x*_1_ ∈ {1, −1}. Then, for every *x*_*j*_, *j* ≠ 1, to be determined, a random number *μ* ∈ (0,1) is generated. Let *x*_*j*_ = *x*_1_ if *μ* < *p*_*j*_^*g*^; otherwise, let *x*_*j*_ = −*x*_1_.

The pseudocode of EDA procedure is given in Algorithm 3. In the procedure, firstly we identify *N* best solutions in population Pop(*g*) (line (1)). The probability vector *p* ^*g*^ is generated according to (6), as well as (7) in line (2). Then, *s* new solutions are generated by the probability vector *p* ^*g*^, and they are further improved by local search procedure (lines (3)–(13)). A path relinking procedure is employed to intensify the search. Line (14) updates ${\hat{x}}^{i}$ and *x* ^*∗*^ if a new better solution is found.

### 2.4. Local Search Procedure

Local search has been proven to be very helpful when incorporated in PSO and EDA [29, 31]. To enhance the exploitation ability, a local search procedure is adopted. It is a simple modification of the local search method (denoted by FMMB) [32] for the max-bisection problem. Experimental results show that the FMMB is very effective. The max-bisection problem consists in partitioning the vertices into two equally sized subsets so as to maximize the sum of the weights of crossing edges. It is a special case of the max-cut problem.

The steps for our local search procedure are presented in Algorithm 4. The local search procedure performs passes repeatedly until a pass fails to generate a better solution. Each pass is described between lines (2) and (19). Let *x* ^best^ be the current best solution found in a pass and let *F* be the set of unlocked vertices. Suppose that *x* ^0^ is a starting solution and its corresponding partition is (*V*_1_, *V*_2_). A pass progresses in epochs. At the beginning of a pass, all vertices are unlocked (i.e., are free to be moved). We move free vertices according to their gains. The gain *g*_*j*_ of a vertex *j* is the objective function value and would increase if vertex *j* is moved from its current belonged subset to the other. More formally,

(8) $$\begin{array}{c} g_{j}=\left\{\begin{array}{cc} \underset{\left\{j,k\right\}\in E,k\in V_{1}}{\sum }w_{jk}-\underset{\left\{j,k\right\}\in E,k\in V_{2}}{\sum }w_{jk}, & j\in V_{1};\\ \underset{\left\{j,k\right\}\in E,k\in V_{2}}{\sum }w_{jk}-\underset{\left\{j,k\right\}\in E,k\in V_{1}}{\sum }w_{jk}, & j\in V_{2}. \end{array}\right. \end{array}$$

Line (4) calculates the gains of all free vertices according to (8). There are two steps in each epoch. An epoch consists of lines (6)–(17). Firstly, the local search procedure moves an unlocked vertex with the highest gain in *F* from its current belonged subset (denoted by *V*_*c*_) to the other subset (denoted by *V*_*o*_). And the current moved vertex is not allowed to be moved again during this pass. Line (8) updates the gains of the affected vertices. Then, an unlocked vertex with the highest gain in *V*_*o*_ is moved to *V*_*c*_. It is locked in this pass. The gains of the affected vertices are updated. To speed up our local search procedure, a pass ends if *n*/10 epochs have been performed. The best partition *x* ^best^ observed during the pass is returned. Then, another pass starts with *x* ^0^ = *x* ^best^. The local search procedure terminates when a pass fails to find a better solution.

### 2.5. Path Relinking Procedure

Path relinking is originally introduced in [33]. It explores trajectories that connect initiating solutions and guiding solutions to find better solutions. Our path relinking procedure uses the current best solution *x* ^*∗*^ as the guiding solution.  Algorithm 5 presents the path relinking procedure in detail. Suppose that *x* is an initiating solution, which is generated by the PSO procedure or the EDA procedure. Given two solutions *x* ^1^ and *x* ^2^, the difference set Δ(*x* ^1^, *x* ^2^) between *x* ^1^ and *x* ^2^ is defined as

(9) $$\begin{array}{c} \Delta \left(\;x^{1},x^{2}\;\right)=\left\{\;j:x_{j}^{1}\neq x_{j}^{2}\;\right\}. \end{array}$$

The distance *d*(*x* ^1^, *x* ^2^) between *x* ^1^ and *x* ^2^ is defined as the number of flipping variables for transforming *x* ^1^ to *x* ^2^. More formally,

(10) $$\begin{array}{c} d\left(\;x^{1},x^{2}\;\right)=\overset{n}{\underset{j=1}{\sum }}\frac{\left|x_{j}^{1}-x_{j}^{2}\right|}{2}. \end{array}$$

Notice that the solution space of the max-cut problem is symmetric; that is, *x* and $\bar{x}=-\left(x_{1},\ldots ,-x_{n}\right)$ represent the same partition. In order to reduce the difference set and speed up the path relinking procedure, we set $x=\bar{x}$ when *d*(*x*, *x* ^*∗*^) > *n*/2. The gains of all vertices are calculated according to (8) in line (5). The difference set Δ(*x*, *x* ^*∗*^) is determined (line (6)). In each iteration, a vertex *a* with the highest gain in *V* is identified (line (8)). If flipping *x*_*a*_ will result in finding a better solution than *x* ^*∗*^, we let *x*_*a*_ = −*x*_*a*_ and stop the path relinking procedure (line (10)). Otherwise, a vertex *b* with the highest gain in Δ(*x*, *x* ^*∗*^) is identified (line (12)), and the vertex *b* is moved from its current belonged subset *V*_*c*_ to the other subset *V*_*o*_; that is, *x*_*b*_ is flipped. The gains of the affected vertices are then updated, and *b* is deleted from Δ(*x*, *x* ^*∗*^) (line (13)). After that, vertex *c* with the highest gain in Δ(*x*, *x* ^*∗*^)∩*V*_*o*_ is determined (line (15)). The gains of the affected vertices are updated, and *c* is deleted from Δ(*x*, *x* ^*∗*^) (line (16)). The above process is repeated until a better solution is found or Δ(*x*, *x* ^*∗*^) = *⌀*.

### 2.6. Mutation Procedure

The PSO-EDA uses the personal best position ${\hat{x}}^{i}$ (*i* = 1,…, *s*) and the current best solution *x* ^*∗*^ found so far to guide the search. At the beginning of the search, the degrees of the similarity between ${\hat{x}}^{i}$ and *x* ^*∗*^ are relatively small, which guides the search to find good solutions quickly. However, with progress of the search, the degrees of the similarity between ${\hat{x}}^{i}$ and *x* ^*∗*^ become large. It makes the search to find a better solution hard.

To make the search retain in a long term, we apply a simple mutation procedure to ${\hat{x}}^{i}$. It diversifies the search. The mutation procedure flips a variable with a probability *κ* = 0.2. In other words, for every ${\hat{x}}_{j}^{i}$, a random number *μ* ∈ (0,1) is generated. The mutation procedure set ${\hat{x}}_{j}^{i}=-{\hat{x}}_{j}^{i}$ if *μ* < *κ*.

## 3. Computational Results and Analysis

In this section, we report the computational experiments to show the efficiency and effectiveness of the PSO-EDA. The PSO-EDA was programmed in C and the experiments were run on PC with AMD processor (3.4 GHz CPU and 4 GB RAM).

### 3.1. Test Instances and Parameter Settings

We use two sets of benchmark instances to test the PSO-EDA. They have been used to test many algorithms for the max-cut problem and max-bisection problem in the last two decades. The first set is G-set graphs [34]. The second set is from [4]. The instances of the second set arise from Ising Spin glasses cubic lattice graphs.

There are several parameters in our proposed PSO-EDA. The values of the population size *s* and the learning speed *α* and *N* and *p*_min_ and *p*_max_ highly affect the performance of PSO-EDA. To investigate the influence of those parameters on the performance of PSO-EDA, we fixed Maxcount = 100, *G*_no_ = 6, prob_*p*_ = 0.25, prob_*n*_ = 0.05, and prob_*r*_ = 0.7 and implemented the Taguchi method of design of experiment (DOE) [31, 35] by using problem G59. Combinations of different values of those parameters are given in Table 1.

For each parameter combination, we run PSO-EDA 5 times independently. We use the orthogonal array *L*_16_(4^4^) and the orthogonal array and the obtained average cut values and average CPU time (time) are listed in Table 2.

From Table 2, one can observe that the PSO-EDA with the third parameter combination (i.e., *s* = 10, *α* = 0.3, *N* = 3, *p*_min_ = 0.2, and *p*_max_ = 0.8) performed better than other parameter combinations in terms of average solution quality and solution time. In the following experiments, the values of parameters in PSO-EDA are given in Table 3.

### 3.2. Comparison of the PSO-EDA with Existing PSO-Based and EDA-Based Algorithms

In this subsection, we compared PSO-EDA with three PSO-based and EDA-based algorithms, that is, a memetic algorithm with genetic particle swarm optimization and neural network (GPSO-CDHNN) [10], a discrete Hopfield network with estimation of distribution algorithm (DHNN-EDA) [25], and tabu Hopfield neural network with estimation of distribution algorithm (THNN-EDA) [9].

We have run PSO-EDA 10 times with parameters listed in Table 3 on some instances used in [9, 25]. Tables 4 and 5 list the best objective function value (*f*_best_), the average objective function value (*f*_avg_), standard deviation values (Std.), and average time (time) in seconds produced by the GPSO-CDHNN, DHNN-EDA, THNN-EDA, and PSO-EDA, respectively. The mark “—” means that the experimental result is not reported. The best objective function value for each selected instance obtained by these algorithms has been indicated in boldface in Tables 4 and 5. The average objective function value with italic indicates the best average objective function value obtained by all algorithms.

The detailed results of GPSO-CDHNN shown in Table 4 are taken from [10]. The data of DHNN-EDA and THNN-EDA is from [9]. Both DHNN-EDA and THNN-EDA were terminated within the same run time, which is shown in the subcolumn “time” under the column “THNN-EDA.” Note that GPSO-CDHNN was tested on DELL-PC (Pentium 4 2.80 GHz), and DHNN-EDA and THNN-EDA were run on a PC (Pentium 4 2.9 GHz with 2.0 G of RAM). According to the CPU speed data from http://www.cpubenchmark.net/, their computers are 6.15 times slower than our computer. Considering the difference between their computers and our computer, we normalize the CPU times of GPSO-CDHNN, DHNN-EDA, and THNN-EDA by dividing them by 6.15.

From Table 4, we observe that PSO-EDA is able to find better solutions compared to GPSO-CDHNN for 14 instances out of 15 selected instances from the first set. In addition, the average objective function values of PSO-EDA are better compared to GPSO-CDHNN for all tested instances from the first set. The CPU time of PSO-EDA is smaller than that of GPSO-CDHNN. These mean that PSO-EDA has a better performance than GPSO-CDHNN.

From Tables 4 and 5, we can see that the best objective function value and the average objective function value of PSO-EDA are much better than those of DHNN-EDA for all 24 considered instances from the first set, as well as 20 instances from the second set. The PSO-EDA takes less CPU time compared to DHNN-EDA for all tested instances, expect for G1, G2, and G3. Therefore, PSO-EDA significantly outperforms DHNN-EDA for these instances.

THNN-EDA and PSO-EDA found the best objective function values on 23 and 40 out of the total 44 tested instances, respectively. The average objective function value of PSO-EDA is better compared to THNN-EDA for 13 instances from the first set, while it fails to match the average results of THNN-EDA for 6 instances from the first set. PSO-EDA is able to find better average results than THNN-EDA for all instances from the second set. The PSO-EDA takes less CPU time compared to THNN-EDA for all tested instances, expect for G1, G2, and G3. These observations reveal that PSO-EDA performs better than THNN-EDA.

From all the results mentioned above, we can conclude that the performance of PSO-EDA is much better than the existing PSO-based and EDA-based algorithms for the max-cut problem.

### 3.3. Comparison of the PSO-EDA with Other Metaheuristic Algorithms

In this subsection, the PSO-EDA is compared with several metaheuristic algorithms for the max-cut problem, including grasp based heuristic (GRASP-TS/PM) [6], path relinking based heuristic (PR2) [11], breakout local search (BLS) [12], and tabu search based hybrid evolutionary algorithm (TSHEA) [13]. To compare PSO-EDA with these state-of-the-art algorithms, the maximum generation Maxcount is increased to 2000. We run PSO-EDA 10 times. Tables 6 and 7 report the best known solutions (Best), the best values (*f*_best_), and average solution values (*f*_avg_) obtained by GRASP-TS/PM, PR2, BLS, TSHEA, and PSO-EDA, respectively. Since GRASP-TS/PM and BLS do not report their results on the instances of the second set, we do not include comparisons with GRASP-TS/PM and BLS on the instances of the second set. The mark “—” in Tables 6 and 7 means that the experimental result is not reported. The subcolumn “gap” under the column “PSO-EDA” lists the deviation of the best solution value obtained by PSO-EDA with respect to the best known solution value Best. The deviation is calculated as follows: ((Best − *f*_best_)/Best) × 100.

Since GRASP-TS/PM, PR2, BLS, TSHEA, and PSO-EDA were coded on different programming languages and run on different hardware platforms, it is very difficult to make a completely fair comparison of the computing time. Therefore, similar to [13], we only compare algorithms based on the solution quality.

We can make the following observations on the results in Tables 6 and 7:

1. Table 6 shows that GRASP-TS/PM, PR2, BLS, TSHEA, and PSO-EDA find the best known solutions on 25, 36, 48, 54, and 43 instances out of the first 54 small or medium instances from the first set, respectively. For 17 large instances from the first set, BLS and TSHEA find the best known solutions on 5 and 13 instances, respectively. The experimental results in Tables 6 and 7 show that BLS and TSHEA are the best performing algorithms.
2. Compared with GRASP-TS/PM and PR2, PSO-EDA finds very competitive results on the first 54 small or medium instances from the first set. In terms of best solution quality and average solution quality, PSO-EDA is better than PR2 on 15 large instances from the first set.

For 20 instances from the second set, in terms of best solution quality, PSO-EDA is better than PR2 on 9 instances and same as PR2 on 11 instances. In terms of average solution quality, PSO-EDA is better than PR2 on 15 instances, same as PR2 on 3 instances, and worse than PR on 2 instances.

(3) PSO-EDA finds the best known solutions on 62 instances out of total 91 instances. In addition to the other 29 instances, PSO-EDA can obtain the best solution with very small deviations to the best known solutions. The range of deviations is only from 0.01% to 0.47%.
(4) For the large scale instances, the performance of PSO-EDA is not stable. Two main reasons are as follows: (I) with the increase of the instance size, the number of the local optima increases rapidly and (II) the degree of similarity between high quality solutions is generally very large [30].

The above computational results show that the proposed algorithm is very effective for solving the max-cut problem.

## 4. Conclusions

We have presented an integrated method based on particle swarm optimization and estimation of distribution algorithm (PSO-EDA) for the max-cut problem. It utilized both the global information and local information. A fast local search procedure was employed to enhance the performance of PSO-EDA. In addition, a path relinking procedure was developed to intensify the search. These strategies achieve a good balance between intensification and diversification.

Two sets of benchmark instances were used to test the performance of PSO-EDA. The comparison of PSO-EDA with the counterpart algorithms in the literatures, including GPSO-CDHNN, DHNN-EDA, and THNN-EDA, shows that PSO-EDA significantly outperforms these algorithms in terms of solution quality and solution time. We also compared our PSO-EDA with other existing metaheuristic algorithms, including GRASP-TS/PM, PR2, BLS, and TSHEA. The computational results showed that the PSO-EDA is able to find high quality solutions on these tested instances. In future work, we look forward to apply this approach to other combinatorial optimization problems.

## Figures and Tables

**Algorithm 1:**
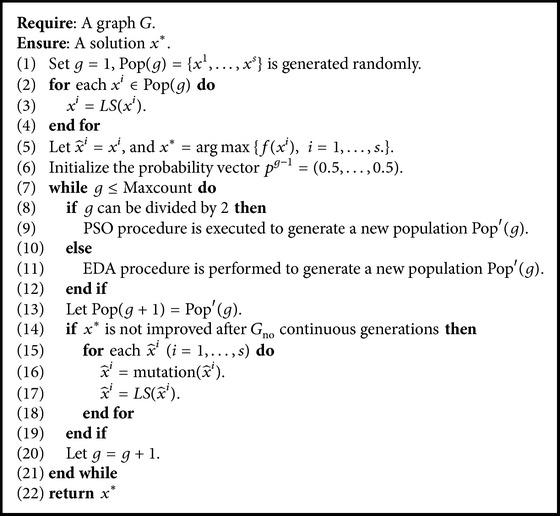
General structure of PSO-EDA.

**Algorithm 2:**
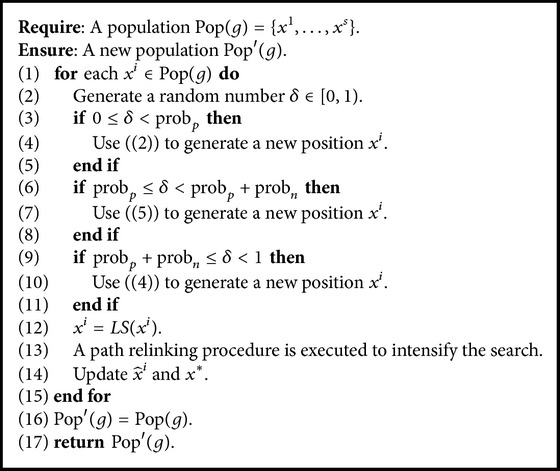
PSO procedure.

**Algorithm 3:**
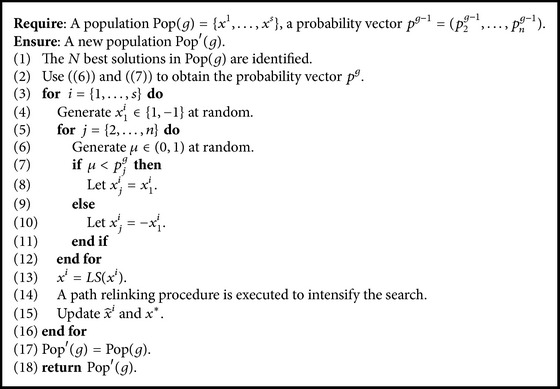
EDA procedure.

**Algorithm 4:**
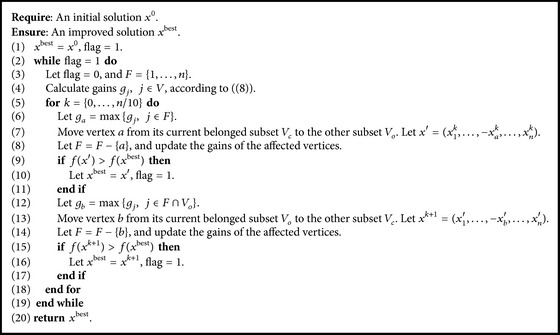
Local search procedure.

**Algorithm 5:**
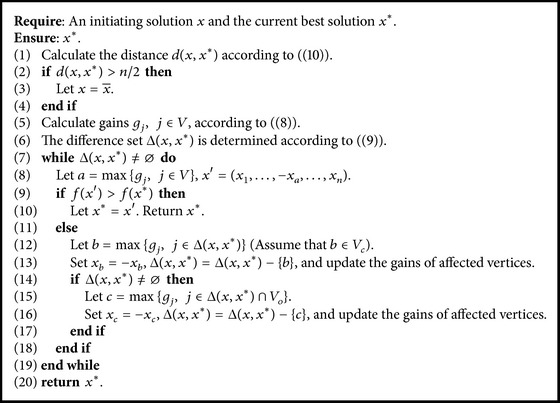
Path relinking procedure.

**Table 1:** Combinations of parameter values.

Parameters	Factor level			
	1	2	3	4
*s*	10	15	20	25
*α*	0.1	0.2	0.3	0.4
*N* (% *s*)	10	20	30	40
(*p*_min_, *p*_max_)	(0.1, 0.9)	(0.15, 0.85)	(0.2, 0.8)	(0.25, 0.75)

**Table 2:** Orthogonal array and average cut values.

Experiment number	Factor				Average cut values	Time
	*s*	*α*	*N* (% *s*)	(*p*_min_, *p*_max_)		
1	1	1	1	1	6023.2	114.344
2	1	2	2	2	6023.0	109.912
3	1	3	3	3	6028.6	107.587
4	1	4	4	4	6008.6	101.788
5	2	1	2	3	6026.8	202.359
6	2	2	1	4	6010.8	186.061
7	2	3	4	1	6019.6	163.295
8	2	4	3	2	6025.0	161.659
9	3	1	3	4	6007.6	289.387
10	3	2	4	3	6014.8	266.276
11	3	3	1	2	6025.4	281.829
12	3	4	2	1	6020.4	267.050
13	4	1	4	2	6024.6	371.903
14	4	2	3	1	6026.4	398.358
15	4	3	2	4	6017.4	376.975
16	4	4	1	3	6026.2	434.746

**Table 3:** Settings of parameters.

Parameters	Section	Values
*s*	2.1	10
*G*_no_	2.1	6
Maxcount	2.1	100
prob_*p*_	2.2	0.25
prob_*n*_	2.2	0.05
prob_*r*_	2.2	0.7
*α*	2.3	0.3
*N*	2.3	3
*p*_min_	2.3	0.2
*p*_max_	2.3	0.8

**Table 4:** Comparison of the results obtained by the GPSO-CDHNN, DHNN-EDA, THNN-EDA, and PSO-EDA on instances from the first set.

Instances	GPSO-CDHNN [10]			DHNN-EDA [25]			THNN-EDA [9]				PSO-EDA			
	*f*_best_	*f*_avg_	time	*f*_best_	*f*_avg_	Std.	*f*_best_	*f*_avg_	Std.	time	*f*_best_	*f*_avg_	Std.	time
G1	—	—	—	11614	11580.6	16.58	**11624**	11621.6	6.9	9.76	**11624 **	* 11624.0 *	0.0	23.714
G2	—	—	—	11599	11584.3	10.22	**11620**	11612.6	7.0	9.76	**11620**	* 11613.3 *	4.84	24.067
G3	—	—	—	11617	11582.5	20.50	**11622**	11619.5	2.6	9.76	**11622**	*11620.0 *	2.45	24.091
G11	**564**	562.56	9.51	494	476.9	5.41	**564**	563.7	0.7	9.76	**564**	* 564.0 *	0.0	2.281
G12	556	554.4	9.46	476	464.7	7.07	**556**	554.7	0.9	9.76	**556**	* 556.0 *	0.0	2.378
G13	580	579.92	9.30	520	501.9	7.35	** 582**	579.8	1.4	9.76	**582**	*582.0 *	0.0	2.273
G14	3058	3057.43	9.47	3027	3017.3	3.86	**3061**	3055.5	3.3	9.76	**3061**	* 3057.3 *	2.58	4.296
G15	3047	3046.67	9.90	2988	2980.9	2.67	**3050 **	3043.2	4.5	9.76	**3050**	* 3045.4 *	3.84	4.521
G16	—	—	—	3001	2991.1	5.72	** 3052**	3043.0	3.5	9.76	**3052**	*3045.2 *	4.54	4.003
G20	940	939.58	9.90	—	—	—	—	—	—	—	**941**	*941.0 *	0.0	3.702
G21	928	926.65	9.79	—	—	—	—	—	—	—	**931**	* 927.9 *	1.60	3.643
G22	13346	13344.4	56.70	13318	13271.7	19.34	**13359**	*13354.0 *	9.7	65.04	**13359**	13347.4	9.97	28.986
G23	13323	13321.3	58.70	13306	13273.6	19.45	**13344**	*13337.8 *	4.0	65.04	13339	13327.6	9.36	28.747
G24	13329	13321.8	58.72	13296	13265.7	16.48	** 13337 **	*13333.7 *	2.6	65.04	**13337**	13323.0	9.36	30.084
G30	3405	3394.62	56.90	—	—	—	—	—	—	—	**3413**	*3407.5 *	5.19	29.905
G31	3293	3290.73	58.65	—	—	—	—	—	—	—	**3306**	*3298.0 *	5.27	33.429
G32	1392	1391.86	56.52	1218	1198.8	9.43	1408	1406.1	1.6	65.04	**1410**	*1406.6 *	1.90	6.304
G33	1368	1367.58	58.41	1200	1160.7	10.69	**1382 **	*1377.2 *	2.0	65.04	**1382**	1377.0	3.16	6.236
G34	1370	1367.57	58.58	1180	1158.2	8.46	**1384 **	* 1380.8*	1.9	65.04	**1384**	*1380.8 *	1.69	6.453
G35	—	—	—	7528	7512.5	7.41	**7680 **	* 7670.0 *	3.7	65.04	7667	7658.2	6.91	17.696
G36	—	—	—	7532	7518.3	5.75	**7671 **	* 7663.3*	4.7	65.04	7657	7643.7	6.81	17.988
G37	—	—	—	7533	7525.8	3.28	**7687**	* 7677.5*	6.6	65.04	7672	7657.8	9.05	17.953
G43	—	—	—	6655	6625.9	13.26	**6660**	6658.0	2.5	19.51	**6660**	* 6658.3 *	1.89	8.556
G44	—	—	—	6641	6623.0	11.23	**6650**	6644.6	4.3	19.51	**6650**	* 6650.0 *	0.0	8.268
G45	—	—	—	6633	6616.1	11.49	**6654 **	6646.3	6.0	19.51	**6654**	*6648.6 *	2.46	8.758
G48	—	—	—	6000	5899.3	58.02	**6000**	*6000.0 *	0.0	9.76	**6000 **	* 6000.0 *	0.0	7.799
G49	—	—	—	6000	5928.2	41.79	**6000 **	*6000.0 *	0.0	9.76	**6000**	* 6000.0 *	0.0	7.892
G50	—	—	—	5868	5815.0	29.40	**5880 **	* 5880.0*	0.0	9.76	**5880**	* 5880.0 *	0.0	7.653

**Table 5:** Comparison of the results obtained by the DHNN-EDA, THNN-EDA, and PSO-EDA on instances from the second set.

Instances	DHNN-EDA [25]			THNN-EDA [9]				PSO-EDA			
	*f*_best_	*f*_avg_	Std.	*f*_best_	*f*_avg_	Std.	time	*f*_best_	*f*_avg_	Std.	time
3dl101000	804	779.93	7.97	894	890.73	3.44	19.51	**896 **	* 892.8 *	3.01	2.971
3dl102000	802	789.13	5.79	**900 **	898.00	2.07	19.51	**900**	* 899.2 *	1.40	2.802
3dl103000	790	778.33	6.02	**892**	887.00	3.30	19.51	**892**	*892.0 *	0.0	2.776
3dl104000	810	796.47	6.84	**898**	896.27	1.61	19.51	**898**	*897.6 *	0.84	2.794
3dl105000	804	787.27	8.27	**886**	882.20	1.81	19.51	**886**	*883.8 *	1.48	2.834
3dl106000	802	797.13	3.25	**888**	884.67	3.03	19.51	**888**	*886.2 *	2.57	2.765
3dl107000	790	783.13	4.61	898	895.40	1.94	19.51	** 900**	* 896.8 *	2.53	2.772
3dl108000	794	769.47	8.94	**882**	877.93	2.28	19.51	**882**	*880.0 *	0.94	2.961
3dl109000	808	797.33	5.47	**902**	897.20	3.12	19.51	**902**	*899.6 *	2.46	2.849
3dl1010000	812	793.87	9.76	**894**	889.67	2.74	19.51	**894**	*891.4 *	1.65	2.770
3dl141000	2176	2137.87	14.89	2438	2429.27	5.62	97.56	**2442**	* 2436.2 *	3.58	11.827
3dl142000	2192	2155.00	16.09	2454	2440.33	6.63	97.56	**2458**	* 2452.4 *	3.63	11.671
3dl143000	2148	2128.27	10.02	2434	2427.07	4.70	97.56	**2438**	* 2433.0 *	3.30	11.923
3dl144000	2180	2147.73	11.85	**2442**	2433.93	5.64	97.56	**2442**	* 2439.4 *	2.50	12.077
3dl145000	2128	2097.93	11.55	2440	2428.93	5.72	97.56	**2446**	*2437.8 *	3.46	12.012
3dl146000	2176	2151.40	10.25	**2448**	2437.73	4.91	97.56	**2448**	*2441.6 *	3.75	12.101
3dl147000	2166	2147.80	8.90	**2440**	2428.20	5.52	97.56	**2440 **	* 2435.6 *	3.37	11.892
3dl148000	2140	2106.87	13.80	2442	2432.60	5.14	97.56	**2448**	* 2440.2 *	3.94	12.922
3dl149000	2112	2090.40	9.80	2420	2412.40	5.30	97.56	**2422**	* 2416.8 *	4.64	11.975
3dl1410000	2172	2150.07	9.80	**2452**	2440.73	5.17	97.56	**2452**	* 2442.8 *	4.34	12.515

**Table 6:** Comparison of the results obtained by the GRASP-TS/PM, PR2, BLS, TSHEA, and PSO-EDA on instances from the first set.

Instances	*n*	Best	GRASP-TS/PM [6]		PR2 [11]		BLS [12]		TSHEA [13]		PSO-EDA		
			*f*_best_	*f*_avg_	*f*_best_	*f*_avg_	*f*_best_	*f*_avg_	*f*_best_	*f*_avg_	*f*_best_	*f*_avg_	Gap (%)
G1	800	11624	11624	11624.0	11624	11624.0	11624	11612.4	11624	11624.0	11624	11624.0	0
G2	800	11620	11620	11620.0	11620	11620.0	11620	11615.0	11620	11620.0	11620	11614.0	0
G3	800	11622	11620	11620.0	11620	11620.0	11622	11621.1	11622	11622.0	11622	11622.0	0
G4	800	11646	11646	11646.0	11646	11646.0	11646	11642.8	11646	11646.0	11646	11642.3	0
G5	800	11631	11631	11631.0	11631	11631.0	11631	11631.0	11631	11631.0	11631	11631.0	0
G6	800	2178	2178	2177.9	2178	2178.0	2178	2178.0	2178	2178.0	2178	2178.0	0
G7	800	2006	2006	2006.0	2006	2006.0	2006	2001.05	2006	2006.0	2006	2006.0	0
G8	800	2005	2005	2004.9	2005	2005.0	2005	2004.4	2005	2005.0	2005	2002.7	0
G9	800	2054	2054	2053.6	2054	2054.0	2054	2049.95	2054	2054.0	2054	2047.1	0
G10	800	2000	2000	1999.3	2000	1999.8	2000	1996.05	2000	2000.0	2000	1997.5	0
G11	800	564	564	564.0	564	564.0	564	564.0	564	564.0	564	564.0	0
G12	800	556	556	556.0	556	556.0	556	556.0	556	556.0	556	556.0	0
G13	800	582	582	581.8	582	582.0	582	582.0	582	582.0	582	582.0	0
G14	800	3064	3063	3062.1	3064	3062.6	3064	3062.85	3064	3064.0	3062	3060.4	0.03
G15	800	3050	3050	3049.1	3050	3049.3	3050	3050.0	3050	3050.0	3050	3047.6	0
G16	800	3052	3052	3050.9	3052	3051.4	3052	3051.1	3052	3052.0	3052	3051.4	0
G17	800	3047	3047	3045.8	3047	3046.4	3047	3046.7	3047	3047.0	3047	3045.1	0
G18	800	992	992	992.0	992	992.0	992	991.7	992	992.0	992	990.5	0
G19	800	906	906	906.0	906	906.0	906	904.55	906	906.0	906	904.4	0
G20	800	941	941	941.0	941	941.0	941	941.0	941	941.0	941	941.0	0
G21	800	931	931	930.6	931	931.0	931	930.2	931	931.0	931	930.3	0
G22	2000	13359	13349	13342.4	13359	13354.5	13359	13344.45	13359	13359.0	13359	13353.1	0
G23	2000	13344	13332	13322.4	13342	13331.6	13344	13340.6	13344	13344.0	13344	13331.8	0
G24	2000	13337	13324	13317.3	13333	13325.3	13337	13329.8	13337	13337.0	13337	13325.1	0
G25	2000	13340	13326	13318.1	13339	13328.2	13340	13333.4	13340	13340.0	13338	13324.2	0.01
G26	2000	13328	13313	13303.3	13326	13312.3	13328	13320.0	13328	13328.0	13326	13319.1	0
G27	2000	3341	3325	3318.1	3336	3326.9	3341	3332.25	3341	3341.0	3341	3323.7	0
G28	2000	3298	3287	3277.4	3296	3288.9	3298	3293.85	3298	3298.0	3298	3290.3	0
G29	2000	3405	3394	3384.5	3405	3391.9	3405	3388.2	3405	3405.0	3405	3388.5	0
G30	2000	3413	3402	3393.4	3411	3404.8	3412	3404.85	3413	3413.0	3412	3405.4	0.03
G31	2000	3310	3299	3287.7	3306	3299.5	3309	3305.3	3310	3310.0	3308	3301.3	0.06
G32	2000	1410	1406	1397.3	1410	1404.6	1410	1409.3	1410	1410.0	1410	1408.6	0
G33	2000	1382	1374	1369.1	1382	1376.1	1382	1380.1	1382	1382.0	1382	1380.4	0
G34	2000	1384	1376	1372.5	1384	1378.2	1384	1384.0	1384	1384.0	1384	1383.2	0
G35	2000	7687	7661	7657.4	7679	7670.8	7684	7680.85	7687	7685.6	7685	7673.5	0.03
G36	2000	7680	7660	7652.1	7671	7658.7	7678	7673.6	7680	7677.5	7671	7660.2	0.12
G37	2000	7691	7670	7662.0	7682	7667.9	7689	7685.85	7691	7688.05	7678	7668.2	0.17
G38	2000	7688	7670	7659.8	7682	7670.4	7687	7684.95	7688	7688.0	7688	7670.8	0
G39	2000	2408	2397	2387.1	2407	2391.1	2408	2405.35	2408	2408.0	2408	2396.7	0
G40	2000	2400	2397	2384.3	2399	2383.3	2400	2394.6	2400	2399.6	2395	2385.3	0.21
G41	2000	2405	2398	2383.7	2404	2388.9	2405	2403.0	2405	2405.0	2405	2387.8	0
G42	2000	2481	2474	2461.7	2478	2466.2	2481	2475.4	2481	2478.45	2478	2470.6	0.12
G43	1000	6660	6660	6659.4	6660	6659.9	6660	6658.15	6660	6659.0	6660	6658.7	0
G44	1000	6650	6649	6647.7	6650	6649.9	6650	6647.7	6650	6650.0	6650	6649.4	0
G45	1000	6654	6654	6652.6	6654	6653.9	6654	6652.15	6654	6654.0	6654	6650.1	0
G46	1000	6649	6649	6646.0	6649	6648.8	6649	6647.75	6649	6649.0	6649	6646.2	0
G47	1000	6657	6656	6655.4	6657	6656.8	6657	6654.35	6657	6657.0	6657	6650.8	0
G48	3000	6000	6000	6000.0	6000	6000.0	6000	6000.0	6000	6000.0	6000	6000.0	0
G49	3000	6000	6000	6000.0	6000	6000.0	6000	6000.0	6000	6000.0	6000	6000.0	0
G50	3000	5880	5880	5880.0	5880	5880.0	5880	5879.9	5880	5880.0	5880	5880.0	0
G51	1000	3848	3847	3843.8	3848	3846.4	3848	3847.85	3848	3848.0	3848	3844.6	0
G52	1000	3851	3850	3846.8	3851	3848.4	3851	3850.85	3851	3851.0	3851	3845.5	0
G53	1000	3850	3848	3845.8	3850	3847.7	3850	3849.5	3850	3849.55	3850	3845.1	0
G54	1000	3852	3850	3847.8	3851	3847.8	3852	3850.6	3852	3851.25	3850	3846.1	0.05
G55	5000	10299	—	—	10265	10234.0	10294	10282.4	10299	10291.75	10293	10267.3	0.06
G56	5000	4017	—	—	3981	3959.2	4012	3998.65	4017	4008.6	4004	3990.0	0.32
G57	5000	3494	—	—	3472	3462.0	3492	3488.6	3494	3488.7	3492	3486.6	0.06
G58	5000	19276	—	—	19205	19182.0	19263	19255.6	19276	19266.0	19251	19213.8	0.13
G59	5000	6085	—	—	6027	6006.2	6078	6067.9	6085	6070.45	6060	6028.2	0.41
G60	7000	14186	—	—	14112	14091.8	14176	14166.8	14186	14173.5	14161	14142.2	0.18
G61	7000	5796	—	—	5730	5695.7	5789	5773.35	5796	5776.0	5769	5756.7	0.47
G62	7000	4868	—	—	4836	4830.2	4868	4863.8	4866	4860.2	4860	4856.2	0.16
G63	7000	27018	—	—	26916	26879.3	26997	26980.7	27018	26993.6	26958	26908.6	0.22
G64	7000	8735	—	—	8641	8594.1	8735	8735.0	8735	8717.95	8710	8672.2	0.29
G65	8000	5560	—	—	5526	5515.9	5558	5551.2	5560	5555.4	5546	5543.4	0.25
G66	9000	6364	—	—	6314	6302.4	6360	6350.2	6364	6353.7	6350	6338.4	0.22
G67	10000	6944	—	—	6902	6884.6	6940	6935.3	6944	6937.3	6932	6927.6	0.17
G70	10000	9548	—	—	9463	9434.0	9541	9527.1	9548	9539.6	9530	9518.4	0.19
G72	10000	6998	—	—	6946	6933.8	6998	6935.3	6990	6979.7	6984	6977.2	0.20
G77	14000	9926	—	—	—	—	9926	9916.1	9902	9890.8	9904	9896.0	0.22
G81	20000	14030	—	—	—	—	14030	14021.7	14010	13993.2	13980	13976.5	0.36

**Table 7:** Comparison of the results obtained by the PR2, TSHEA, and PSO-EDA on instances from the second set.

Instances	*n*	Best	PR2 [11]		TSHEA [13]		PSO-EDA		
			*f*_best_	*f*_avg_	*f*_best_	*f*_avg_	*f*_best_	*f*_avg_	Gap (%)
3dl101000	1000	896	896	894.6	896	896.0	896	896.0	0
3dl102000	1000	900	900	900.0	900	900.0	900	900.0	0
3dl103000	1000	892	892	891.3	892	892.0	892	892.0	0
3dl104000	1000	898	898	898.0	898	898.0	898	898.0	0
3dl105000	1000	886	886	885.4	886	886.0	886	886.0	0
3dl106000	1000	888	888	888.0	888	888.0	888	888.0	0
3dl107000	1000	900	900	898.2	900	900.0	900	898.8	0
3dl108000	1000	882	882	881.2	882	882.0	882	880.6	0
3dl109000	1000	902	902	901.5	902	902.0	902	902.0	0
3dl1010000	1000	894	894	893.7	894	894.0	894	892.6	0
3dl141000	2744	2446	2444	2437.6	2446	2446.0	2446	2443.0	0
3dl142000	2744	2458	2456	2452.4	2458	2458.0	2458	2455.8	0
3dl143000	2744	2442	2438	2435.5	2442	2442.0	2442	2438.4	0
3dl144000	2744	2450	2448	2440.0	2450	2449.4	2448	2443.2	0.08
3dl145000	2744	2446	2444	2438.7	2446	2446.0	2446	2444.0	0
3dl146000	2744	2452	2448	2442.3	2452	2451.4	2452	2445.8	0
3dl147000	2744	2444	2440	2435.0	2444	2444.0	2444	2439.2	0
3dl148000	2744	2448	2444	2438.9	2448	2447.6	2448	2443.6	0
3dl149000	2744	2428	2422	2417.3	2428	2426.3	2428	2422.4	0
3dl1410000	2744	2460	2454	2448.8	2460	2458.4	2456	2451.8	0.16
